# Evaluating the efficacy of using large language models in preoperative prediction of microvascular invasion in HCC: a multicenter study

**DOI:** 10.1038/s41598-025-08502-4

**Published:** 2025-07-29

**Authors:** Zongren Ding, Jianxing Zeng, Guoxu Fang, Pengfei Guo, Weiping Zhou, Yongyi Zeng

**Affiliations:** 1 Department of Hepatopancreatobiliary Surgery, Mengchao Hepatobiliary Hospital of Fujian Medical University, Jintang Road 66, Fuzhou, China; 2 The Big Data Institute of Southeast Hepatobiliary Health Information, Mengchao Hepatobiliary Hospital of Fujian Medical University, Fuzhou, China; 3 The Third Department of Hepatic Surgery, Eastern Hepatobiliary Surgery Hospital, Second Military Medical University, Shanghai, China

**Keywords:** Hepatocellular carcinoma, Large language models, Microvascular invasion

## Abstract

Primary liver cancer is the sixth most commonly diagnosed cancer globally and the third leading cause of cancer-related deaths. Hepatocellular carcinoma (HCC) is the most common type of primary liver cancer, and microvascular invasion (MVI) is a significant risk factor affecting postoperative prognosis in HCC. However, accurately predicting MVI preoperatively remains a challenge. This study aims to evaluate the application of large language models (LLMs), specifically ChatGPT 4o, in predicting MVI in HCC and to compare its performance with traditional clinical models. In this retrospective study, 300 HCC patients who underwent curative liver resection between June 2018 and December 2018 were selected at two centers. The collected clinical data included age, gender, HBV infection, liver cirrhosis, AFP levels, and more. ChatGPT 4o were used to process the clinical data of the patients and predict MVI. Subsequently, the predictive results of the ChatGPT 4o were compared with machine learning models, the ROC curves were plotted, and AUC was calculated. The results showed that the AUC of the ChatGPT 4o was 0.755. Machine learning algorithms use Random Forest, Support Vector Machine, Logistic Regression, XGBoost and Decision Tree, the AUC of 5 machine learning algorithms was range from 0.534 to 0.624. ChatGPT 4o achieved the highest AUC and showed statistically significant differences compared to Support Vector Machine, Logistic Regression and Decision Tree. Additionally, the predictive results of the ChatGPT 4o effectively stratified the postoperative overall survival (OS) and recurrence-free survival (RFS) of HCC patients. LLMs have demonstrated significant predictive capabilities for MVI in HCC and for risk stratification regarding postoperative OS and RFS. These advancements possess substantial potential to enhance preoperative management and make surgical planning.

**Supplementary Information:**

The online version contains supplementary material available at 10.1038/s41598-025-08502-4.

## Introduction

Primary liver cancer is the sixth most commonly diagnosed cancer globally and the third leading cause of cancer-related deaths, with approximately 906,000 new cases and 830,000 deaths in 2020^1^. Primary liver cancer includes hepatocellular carcinoma (HCC), intrahepatic cholangiocarcinoma, and other rare types, with HCC being the most common. The main risk factors for HCC including chronic infection with hepatitis B virus (HBV) or hepatitis C virus (HCV), heavy alcohol consumption, and being overweight^2^. Due to the high prevalence of HBV in China, the country is considered a high-risk area for HCC, resulting in a significant socioeconomic burden from HCC^3^.

Liver resection may offer a curative option for patients with early-stage HCC^4,5^. However, even after surgical resection, the recurrence rates at 1, 3, and 5 years are 39.0%, 63.9%, and 74.9%, respectively^6^. The overall survival (OS) rates at 1, 3, and 5 years are 85.9%, 60.8%, and 47.0%, respectively^6^. This indicates that the prognosis after surgery remains unsatisfactory. Therefore, it is necessary to further investigate the risk factors affecting postoperative prognosis.

The biological characteristics of HCC often include the presence of microvascular invasion (MVI)^7^. MVI is an important risk factor affecting the prognosis after surgical resection and is significantly associated with an increased risk of tumor recurrence following liver resection^8^. Studies have shown that HCC patients with MVI can benefit from anatomical liver resection compared to non-anatomical liver resection^9^. Additionally, for patients with a high preoperative risk of MVI, liver resection with a wide margin (≥ 1 cm) has a better prognosis than with a narrow margin^10^. Therefore, accurately predicting MVI preoperatively is of significant reference value for the management of the surgical process. However, it is not possible to determine whether a patient has MVI preoperatively, as its diagnosis can only be obtained from postoperative histopathological examination of surgical specimens.

Currently, many studies have reported non-invasive preoperative prediction of MVI. The maximal variant allele frequency (VAF) of circulating tumor DNA (ctDNA) was used to predict MVI with an AUC (The area under the curve) of 0.85^11^. The DNA methylation signature and the expression level of exosomal PRPSAP1 in plasma have also been shown to have potential in predicting MVI^12,13^. More studies have used clinical variables for prediction. In a study of over 1,000 liver resection samples, a nomogram incorporating seven risk factors, including tumor size, multiple nodules, and alpha-fetoprotein (AFP), achieved the best preoperative prediction of MVI in HBV-related HCC within the Milan criteria, with an AUC of 0.80^14^. Another study with nearly 500 samples developed a nomogram that included five risk factors, such as FIB-4, AFP levels, and cirrhosis, with an AUC of 0.786^15^. Additionally, inflammatory markers such as the gamma-glutamyl transpeptidase to lymphocyte ratio (GLR) and the neutrophil-to-lymphocyte ratio (NLR) have also been shown to be predictors of MVI^16,17^. Preoperative imaging of HCC contains important radiomics features, and radiomics models based on enhanced CT or enhanced MR have also achieved good predictive performance when combined with clinical features, the AUC further improved to 0.889–0.920^18,19^.

Although the aforementioned models have achieved good predictive performance, they have not been widely applied in clinical practice. There are two main reasons for this: first, these models usually involve complex computational processes; second, genomic or radiomic data are often not easily accessible. Therefore, simpler and more efficient predictive tools are urgently needed for broader clinical application.

The emergence of large language models (LLMs) technology seems to offer an opportunity to address this issue. Through pre-training and fine-tuning processes, LLMs can learn complex semantic and contextual relationships from extensive text datasets^20^. This capability makes them particularly advantageous in handling medical text data, extracting latent features, and performing predictive analysis^21^. More importantly, they are very convenient to use, as they operate through conversational interfaces. Previous studies have shown that LLMs have made significant progress in areas such as medical literature retrieval, clinical decision support, and disease prediction^22^. However, the application of LLMs in predicting MVI in HCC remains relatively unexplored.

Therefore, our aim was to use LLMs to process patients’ clinical data, explore its predictive capability for MVI in HCC, and evaluate the model by comparing it with traditional machine learning models.

## Materials and methods

### Patients and clinical characteristics

**Fig. 1:**
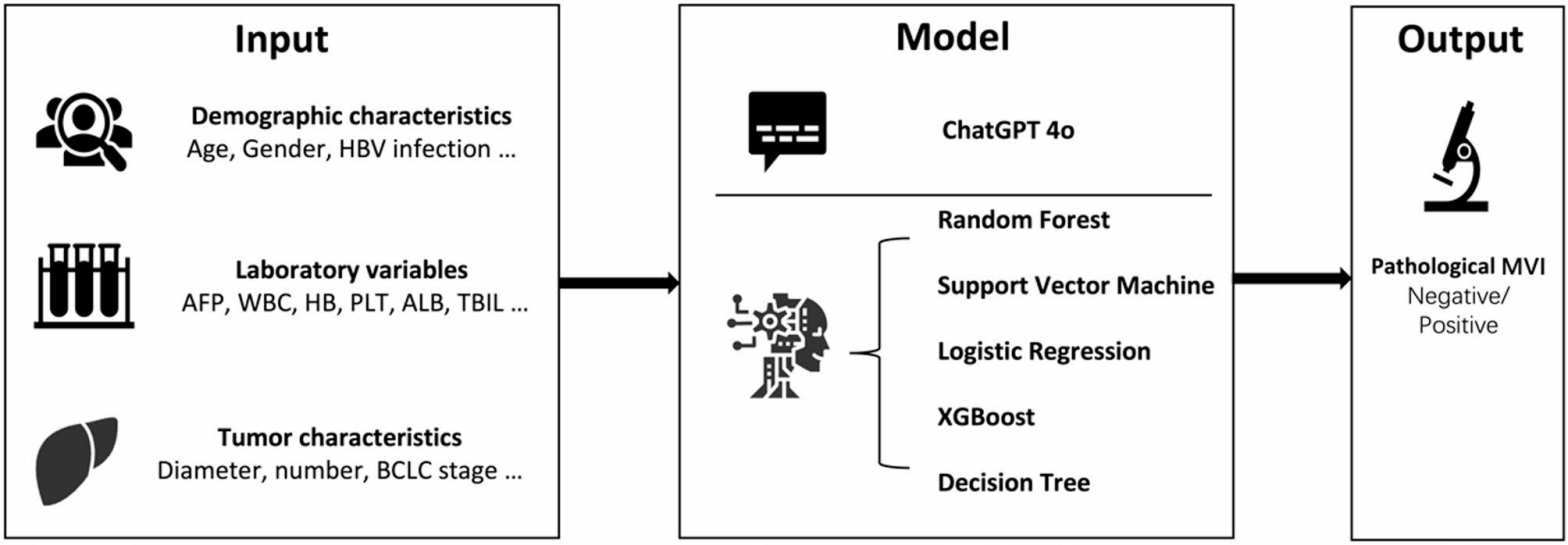
The workflow of this study.

The workflow of this study was shown in Fig. 1. In this multicenter retrospective study, from June 2018 to December 2018, 300 patients with HCC who underwent curative-intent liver resection at the Mengchao Hepatobiliary Hospital of Fujian Medical University and Eastern Hepatobiliary Surgery Hospital were selected. All patients had complete postoperative pathology reports, most importantly including the diagnosis information of MVI. MVI-positive was defined as the presence of cancer cell nests within the lumen of vessels lined by endothelial cells under the microscope^23^.

The collected clinical data included age, gender, HBV (Hepatitis B Virus), liver cirrhosis, AFP (Alpha-Fetoprotein), WBC (White Blood Cell), HB (Hemoglobin), PLT (Platelet), ALB (Albumin), TBIL (Total Bilirubin), GGT (Gamma-Glutamyl Transferase), ALP (Alkaline Phosphatase), diameter of tumor, number of tumors, BCLC (Barcelona Clinic Liver Cancer), and MVI. All patients underwent regular follow-up after surgery, and the RFS (recurrence free survival) and OS (overall survival) was collected.

This study was approved by the Institutional Ethics Committee of Mengchao Hepatobiliary Hospital of Fujian Medical University, and written informed consent was obtained from all participants. The research was conducted in accordance with the ethical standards outlined in the 1964 Declaration of Helsinki and its subsequent amendments, or comparable ethical standards.

### Machine learning model

All cases were divided into training and validation sets in a 7:3 ratio. The training set was used to build the model, while the validation set was used to evaluate the model’s predictive performance. The comparison of the baseline data of the two sets is shown in the supplementary materials, and there is no significant difference in the baseline data of the two sets (*P* > 0.05). The algorithms used included five of the most common machine learning algorithms: Random Forest, Support Vector Machine, Logistic Regression, XGBoost, and Decision Tree. These algorithms were implemented using specific “packages” in the R software.

### Large language models

We chose ChatGPT 4o as the LLM for prediction. Each patient’s clinical data was integrated into a paragraph, followed by the prediction task. It was confirmed that the fields did not contain any personal information or sensitive privacy content.

First, input the patient’s information, and then ask ChatGPT 4o the question. For example:

Patient’s information: A 48-year-old male patient clinically diagnosed with hepatocellular carcinoma, with a history of chronic hepatitis B virus infection, but no liver cirrhosis. Laboratory results showed alpha-fetoprotein (AFP) at 14.2 ng/mL, white blood cells (WBC) at 4.98 * 10^9/L, hemoglobin (HB) at 138 g/L, platelets (PLT) at 162 * 10^9/L, albumin (ALB) at 38.9 g/L, total bilirubin (TBIL) at 9.5 μmol/L, gamma-glutamyl transferase (GGT) at 51 IU/L, and alkaline phosphatase (ALP) at 68 IU/L. Preoperative imaging indicated a single tumor with a maximum diameter of 2.6 cm, no major vascular invasion, and no distant metastasis.

Question: After the patient undergoes radical liver resection, will the pathology results show microvascular invasion (MVI)? Please answer “yes” or “no” and provide the reason.

After all patients completed the Q&A, the prediction results from the ChatGPT 4o were used as risk factors to perform binary logistic regression modeling. The ROC (Receiver operating characteristic) curves was plotted, and the AUC (The area under the curve) was calculated.

### Data privacy and ethical compliance

This study strictly adhered to data privacy and ethical compliance requirements, retaining only clinically relevant variables (including age, gender, HBV infection status, tumor characteristics, laboratory markers, and prognostic outcomes [RFS, OS]), while removing all direct identifiers (e.g., names, ID numbers) and indirect identifiers (e.g., exact birth dates, rare disease diagnoses). For data security, raw data were stored on secure servers at Mengchao Hepatobiliary Hospital of Fujian Medical University with AES-256 encryption, with access restricted to three authorized researchers via two-factor authentication, and all data operations were logged and archived for traceability. Ethically, this retrospective study was approved by the Ethics Committee of Fujian Medical University Mengchao Hepatobiliary Hospital (Approval No. 2021–100-06) and complied with China’s Regulations on Ethical Review of Biomedical Research Involving Humans; due to the fully anonymized nature of the data, the ethics committee waived the requirement for informed consent. To minimize selection bias, the study consecutively included HCC cases (N = 300) treated between June and December 2018, ensuring cohort representativeness.

### Statistical analysis

Statistical analysis was done using R (v4.4.1). Categorical variables were compared using the χ2 test or Fisher exact test. Continuous variables were expressed as the median [Q1, Q3] and compared using the Student T test or Mann–Whitney U test. Delong test was used to measure the differences of the AUC in ROC curves^24^. Kaplan–Meier (KM) curves were used for survival analysis. *P* < 0.05 was considered statistically significant.

## Results

Among all patients in this study, the median age of the patients was 51.0 years. In terms of gender distribution, females accounted for 11.6% and males for 88.4%. Regarding BCLC staging, 6.0% of the patients were in stage 0, and 94.0% were in stage A. The MVI-negative group, consisting of 205 patients, accounted for 68.3%, while the MVI-positive group, consisting of 95 patients, accounted for 31.7% (Table 1).

**Table 1:** Comparison of clinical characteristics between MVI ( −) and MVI ( +) patients.

Clinical characteristics	MVI( −) (N = 205)	MVI( +) (N = 95)	*P*
Age, years	51.0 [44.0, 60.0]	51.0 [43.5, 59.5]	0.536
Gender, Female/Male	28 (13.7%)/177 (86.3%)	7 (7.4%)/88 (92.6%)	0.166
HBV infection, Negative/ Positive	32 (15.6%)/173 (84.4%)	13 (13.7%)/82 (86.3%)	0.794
Liver cirrhosis, Negative/ Positive	82 (40.0%)/123 (60.0%)	40 (42.1%)/55 (57.9%)	0.827
AFP, ng/mL	12.7 [3.70, 424]	123 [6.85, 1210]	**0.015**
WBC, 10^9/L	5.41 [4.74, 6.24]	5.69 [5.01, 6.51]	0.205
HB, g/L	146 [137, 155]	148 [142, 157]	0.078
PLT, 10^9/L	175 [149, 217]	179 [141, 208]	0.465
ALB, g/L	43.0 [41.3, 45.2]	43.5 [41.7, 45.5]	0.887
TBIL, μmol/L	12.7 [10.0, 16.2]	12.4 [10.5, 15.7]	0.615
GGT, IU/L	46.0 [31.0, 74.0]	42.0 [27.0, 68.5]	0.387
ALP, IU/L	72.0 [59.0, 88.0]	69.0 [60.5, 84.0]	0.522
Diameter of tumor, cm	4.30 [2.80, 5.90]	4.10 [3.10, 5.65]	0.469
Number of tumor, Single/Multiple	198 (96.6%)/7 (3.4%)	90 (94.7%)/5 (5.3%)	0.721
BCLC, 0/A	14 (6.8%)/191 (93.2%)	4 (4.2%)/91 (95.8%)	0.531

MVI (Microvascular Invasion); HBV (Hepatitis B Virus); AFP (Alpha-Fetoprotein); WBC (White Blood Cell); HB (Hemoglobin); PLT (Platelet); ALB (Albumin); TBIL (Total Bilirubin); GGT (Gamma-Glutamyl Transferase); ALP (Alkaline Phosphatase); BCLC (Barcelona Clinic Liver Cancer).

Significant values are in [bold].

**Fig. 2:**
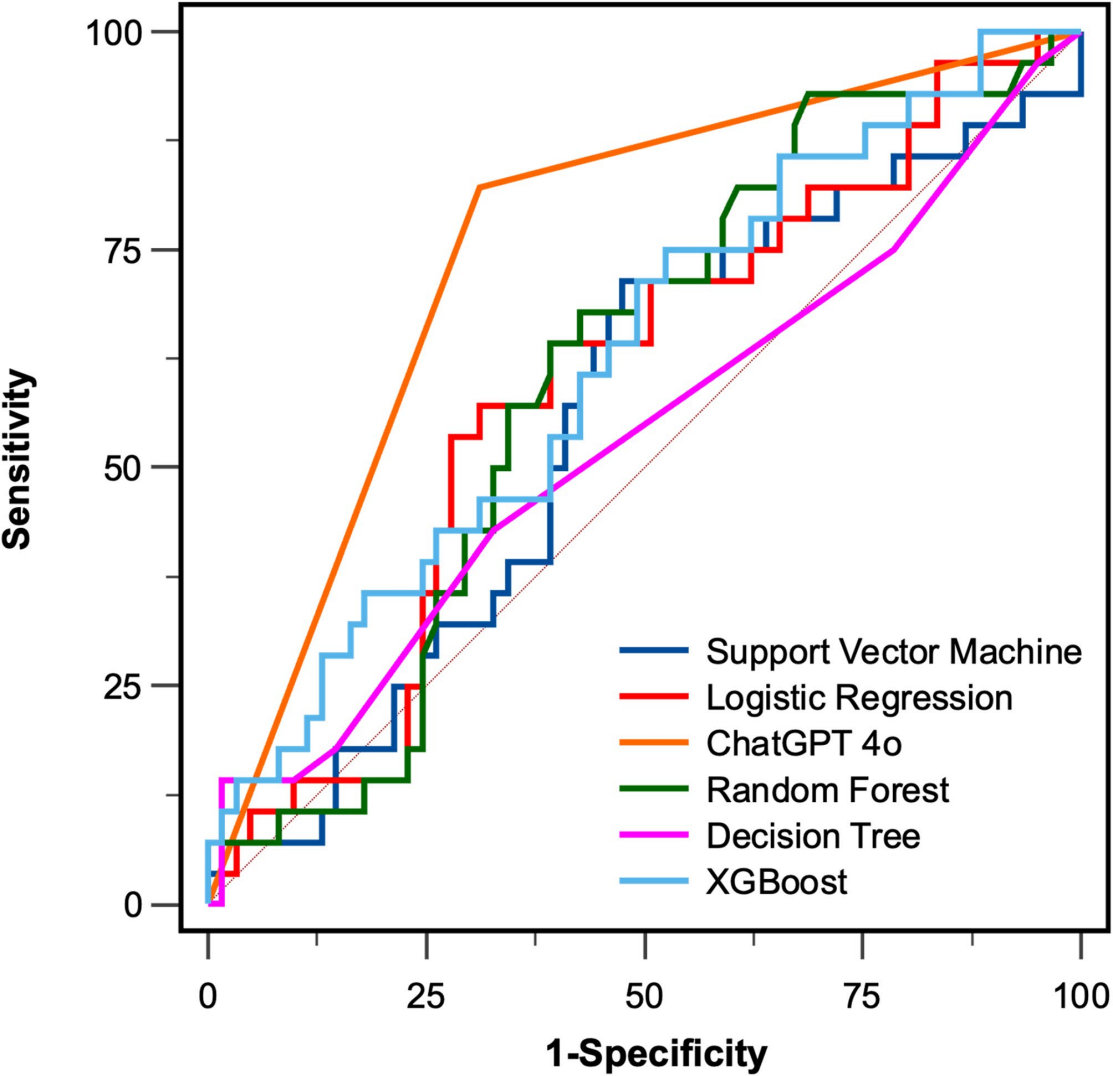
Comparison of ROC Curves of the models. Note: ROC (Receiver operating characteristic); AUC (Area Under the Curve).

**Fig. 3:**
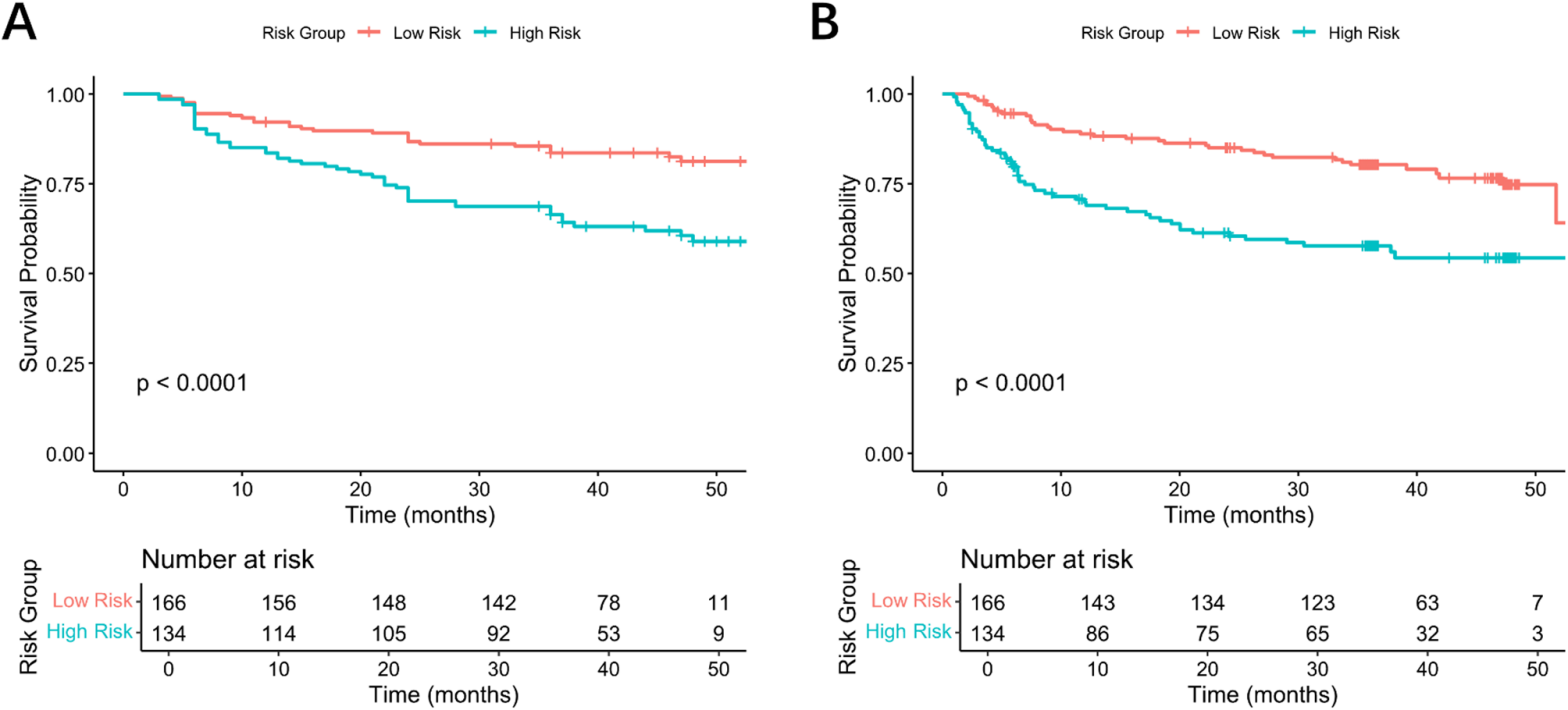
Kaplan–Meier curves for survival analysis of OS (**A**) and RFS (**B**) using the constructed ChatGPT 4o MVI prediction model. Note: OS (overall survival); RFS (recurrence-free survival); MVI (Microvascular Invasion).

The sensitivity, specificity, and AUC of ChatGPT 4o was 0.821(0.631–0.939), 0.689(0.557–0.801), and 0.755(0.652–0.840) (Table 2). As for the 5 machine learning models, XGBoost achieved the highest sensitivity and AUC with 0.750(0.551–0.893) and 0.624(0.515–0.724), Decision Tree achieved the highest specificity with 0.984(0.912–1.000). ChatGPT 4o achieved the highest AUC, and was with statistically significant when compared with Support Vector Machine, Logistic Regression and Decision Tree (Fig. 2). Then, we used the results generated by ChatGPT 4o, which predicted the presence or absence of MVI, as a binary stratification factor (MVI predicted as present was considered high risk, MVI predicted as absent was considered low risk) to perform KM analysis on the survival data of HCC. The results showed that both OS and RFS could achieve significant risk stratification using ChatGPT 4o (*P* < 0.001) (Fig. 3).

**Table 2:** Comparison of predict performance between the models.

	Sensitivity	Specificity	AUC	*P*-value*
ChatGPT 4o	0.821(0.631–0.939)	0.689(0.557–0.801)	0.755(0.652–0.840)	Reference
Random Forest	0.679(0.476–0.841)	0.574(0.441–0.700)	0.605(0.495–0.707)	0.0516
Support Vector Machine	0.714(0.513–0.868)	0.525(0.393–0.654)	0.557(0.448–0.663)	**0.0106**
Logistic Regression	0.571(0.372–0.755)	0.689 (0.557–0.801)	0.597(0.487–0.699)	**0.0449**
XGBoost	0.750(0.551–0.893)	0.475(0.346–0.607)	0.624(0.515–0.724)	0.0802
Decision Tree	0.143(0.040–0.327)	0.984(0.912–1.000)	0.534(0.425–0.640)	**0.0115**

AUC (Area Under the Curve); The values in parentheses were 95% CI (confidence intervals); The *P*-value refers to the direct comparison of AUC, using the DeLong test.

Significant values are in [bold].

## Discussion

In this multicenter study, we used LLMs for predicting MVI in HCC. The results showed that the predictive performance of the LLMs was significantly superior to that of models based on traditional machine learning models. Moreover, the predictions made by the LLMs enabled effective risk stratification of patients. To the best of our knowledge, this is the first study to use LLMs for the prediction of MVI and risk stratification in HCC.

Compared to machine learning and the previous studies reported nomogram and radiomics models, LLMs have an unparalleled advantage: they are simple to operate and easy to implement. More importantly, these models are continuously iterating, potentially taking on more tasks in the future with increasingly better performance. However, several issues were identified during the research process. Firstly, performing similar Q&A at different time points may yield different or even contradictory results. To address this, we conducted multiple tests at different times, typically an odd number of tests, and selected the most frequently occurring result as the final answer. Secondly, it is recommended to perform the same task within a single conversation and avoid unrelated Q&A, as this may affect the results. Finally, the application of LLMs in clinical settings still faces ethical challenges. In the future, regulations or laws need to be established to govern the use of LLMs in clinical practice. Similarly, our study is exploratory in nature and differs from traditional formal clinical research.

When we conducted machine learning modeling, we found that AFP was always the strongest predictor of MVI, which was consistent with previous literature^15,25–27^. However, features such as tumor size and multiple tumors were not included, even though these indicators were typically considered risk factors for MVI^26–28^. This phenomenon might have been due to the data structure; in other words, in our dataset, tumor size and multiple tumors were not risk factors for MVI, whereas in another dataset, the results might have been different. Therefore, machine learning models were highly dependent on the data, and the results were determined by the nature of the data. However, LLMs did not have this issue. During our Q&A process, we found that LLMs always considered more factors. They incorporated all the risk factors for MVI they had learned into their analysis and provided a comprehensive judgment before giving a result. This might have been the most important reason why LLMs outperformed Clinical models in our study.

Our study has some limitations. Firstly, although we included samples from multiple centers, retrospective studies inherently have selection bias. Secondly, our AUC was relatively low compared to the reports in the existing literature, at 0.755, indicating there is room for improvement. In the future, incorporating multimodal data such as images and audio recordings may lead to an improvement in AUC. Thirdly, our study did not compare ChatGPT 4o with biomedical pre-trained models (e.g., BioBERT, PubMedBERT) or other large language models (e.g., Google Gemini, Claude), which may limit a comprehensive evaluation of its relative performance. Future research could incorporate these models as benchmarks to further validate and expand the findings. Lastly, the underlying logic of LLMs involves using deep learning algorithms to predict the next word iteratively, which introduces an element of randomness. Although we attempted multiple queries to mitigate this, the randomness cannot be entirely eliminated. Future technological advancements may lead to more stable responses from LLMs.

## Conclusion

LLMs have exhibited notable predictive capabilities for MVI in HCC, outperforming machine learning models in this regard. Furthermore, the predictive outcomes generated by LLMs facilitated effective risk stratification for postoperative OS and RFS in HCC patients. These advancements hold significant potential for enhancing preoperative management and making surgical planning.

## Electronic supplementary material

Below is the link to the electronic supplementary material.

Supplementary Material 1

## Data Availability

The datasets used and/or analyzed during the current study are available from the corresponding author on reasonable request.
