# Supplementary material for: Evaluating the efficacy of using large language models in preoperative prediction of microvascular invasion in HCC: a multicenter study
Source: Sci Rep. 2025 Jul 29;15:27549. doi: 10.1038/s41598-025-08502-4 (PMC12307873; doi:10.1038/s41598-025-08502-4)
Supplement: Supplementary file 1 — Supplementary Material 1 [file 41598_2025_8502_MOESM1_ESM.docx]

|  | **Training set (N=210)** | **Validation set**  **(N=90)** | **P** |
| --- | --- | --- | --- |
| **Age, years** | 51.0 [43.0, 59.0] | 52.5 [46.0, 61.0] | 0.333 |
| **Gender, Female/Male** | 24 (11.4%)/186 (88.6%) | 11 (12.2%)/79 (87.8%) | 1.000 |
| **BCLC, 0/A** | 14 (6.7%)/196 (93.3%) | 4 (4.4%)/86 (95.6%) | 0.633 |
| **HBV infection, Negative/ Positive** | 31 (14.8%)/179 (85.2%) | 14 (15.6%)/76 (84.4%) | 1.000 |
| **Liver cirrhosis, Negative/ Positive** | 79 (37.6%)/131 (62.4%) | 43 (47.8%)/47 (52.2%) | 0.130 |
| **AFP, ng/mL** | 26.6 [3.80, 558] | 35.4 [6.23, 665] | 0.826 |
| **WBC, 10^9/L** | 5.43 [4.79, 6.23] | 5.58 [4.94, 6.50] | 0.310 |
| **HB, g/L** | 147 [139, 155] | 147 [138, 155] | 0.378 |
| **PLT, 10^9/L** | 175 [148, 211] | 183 [148, 214] | 0.945 |
| **ALB, g/L** | 43.1 [41.3, 45.1] | 43.4 [41.5, 45.7] | 0.643 |
| **TBIL, μmol/L** | 12.4 [10.0, 16.3] | 12.9 [10.7, 15.0] | 0.823 |
| **GGT, IU/L** | 44.0 [27.0, 75.0] | 46.5 [29.3, 68.0] | 0.611 |
| **ALP, IU/L** | 70.5 [59.0, 88.0] | 75.0 [60.5, 85.0] | 0.889 |
| **Diameter of tumor, cm** | 4.20 [2.93, 6.08] | 4.20 [3.10, 5.40] | 0.938 |
| **Number of tumor, Single/Multiple** | 203 (96.7%)/7 (3.3%) | 85 (94.4%)/5 (5.6%) | 0.563 |
| **MVI,Negative/ Positive** | 147 (70.0%)/63 (30.0%) | 58 (64.4%)/32 (35.6%) | 0.416 |

**Supplementary Table 1. Comparison of clinical characteristics between training set
and validation set.** Note: MVI (Microvascular Invasion); HBV (Hepatitis B Virus); AFP (Alpha-Fetoprotein); WBC (White Blood Cell); HB (Hemoglobin); PLT (Platelet); ALB (Albumin); TBIL (Total Bilirubin); GGT (Gamma-Glutamyl Transferase); ALP (Alkaline Phosphatase); BCLC (Barcelona Clinic Liver Cancer)
